# Lateral Flow Immunoassay for Visible Detection of Human Brucellosis Based on Blue Silica Nanoparticles

**DOI:** 10.3389/fvets.2021.771341

**Published:** 2021-12-03

**Authors:** Lirui Ge, Dan Wang, Fengnan Lian, Jinbin Zhao, Yue Wang, Yuyi Zhao, Lanting Zhang, Juan Wang, Xiuling Song, Jinhua Li, Kun Xu

**Affiliations:** ^1^School of Public Health, Jilin University, Changchun, China; ^2^Public Health Detection Engineering Research Center of Jilin Province, Changchun, China

**Keywords:** blue SiNPs, LFIA, human brucellosis, antigen-antibody sandwich structure, *Brucella* spp.

## Abstract

Brucellosis is a highly contagious zoonosis chronic infectious disease with a strong latent capability to endanger human health and economic development *via* direct or indirect ways. However, the existing methods for brucellosis diagnosis are time-consuming and expensive as they require a tedious experimental procedure and a sophisticated experimental device and performance. To overcome these defects, it is truly necessary to establish a real-time, on-site, and rapid detection method for human brucellosis. Here, a lateral flow immunoassay (LFIA) with a rapid, sensitive, and alternative diagnostic procedure for human brucellosis with a high degree of accuracy was developed based on blue silica nanoparticles (SiNPs), *Staphylococcal* protein A (SPA), and surface Lipopolysaccharide of *Brucella* spp. (LPS), which can be applied for rapid and feasible detection of human brucellosis. To our knowledge, this is the first report that uses blue SiNPs as a signal probe of LFIA for the rapid diagnosis of human brucellosis. The precursor of blue SiNPs@SPA such as colorless SiNPs and blue SiNPs was synthesized at first and then coupled with SPA onto the surface of blue SiNPs by covalent bond to prepare blue SiNPs@SPA as a capture signal to catch the antibody in the brucellosis-positive serum. When SPA was combined with the antibodies in the brucellosis-positive serum, it was captured by LPS on the test line, forming an antigen–antibody sandwich structure, resulting in the T line turning blue. Finally, the results showed that it is acceptable to use blue SiNPs as visible labels of LFIA, and standard brucellosis serum (containing *Brucella* spp. antibody at 1,000 IU/ml) could be detected at a dilution of 10^−5^ and the detection limit of this method was 0.01 IU/ml. Moreover, it also demonstrated good specificity and accuracy for the detection of real human serum samples. Above all, the blue SiNPs-based LFIA that we developed provides a rapid, highly accurate, and inexpensive on-site diagnosis of human brucellosis, and shows great promise in clinical diagnostics for other diseases.

## Introduction

A lateral flow immunoassay (LFIA) is a fast, simple, and economic method that is widely used in the field of rapid detection of various analytes. The labels used for LFIA include a series of particles, such as quantum dots (1, 2), latex beads (3), colloidal gold, and magnetic particles (4). Among the above particles, colloidal gold was the most commonly used label for LFIA, which was widely applied in bacteria detection and drug testing. However, the performance of colloidal gold depends on the amounts of molecules gathered. Thus, it is susceptible to optical interference, which always exhibits relatively low sensitivity and inaccurate qualitative or semi-quantitative results (5). Meanwhile, the performance of colloidal gold is often affected by temperature, pH, and salt concentration. Moreover, the use of colloidal gold in multiple tests is also limited as colloidal gold always displays a single color (6). Quantum dots and dye-doped nanoparticles are representative fluorescent nanoparticle probes that have attracted increasing research attention (5, 7, 8). Dye-doped nanoparticles vary in diameter from 2 to 200 nm, containing a large quantity of dye molecules embedded in a polymer or silica matrix, which can emit more intense fluorescence signals than organic fluorophores (5, 9). However, the detection of fluorescent dye-doped nanoparticles requires specific equipment for the detection of the fluorescence signal, and the stability of the fluorescent signal still needs further verification. Of note, silica nanoparticles (SiNPs) are widely used in several fields, such as in disease labeling, biosensing, and drug delivery (10). However, most previously used SiNPs are white or colorless, which are not suitable for signal conversion and signal amplification (11). It is known that organic dyes possess good stability and rich color, which would not fade even under harsh conditions. Due to their chemical reactivity, many organic dyes are very suitable for dyeing, such as dyeing colorless SiNPs into colored SiNPs. Besides, the surface of SiNPs is easy to modify, which can be used in catalysis, molecular detection, biological detection, and imaging by doping functional materials (12, 13). Therefore, colored SiNPs not only possess a bright color but also possess four advanced characteristics: a strong covalent interaction between dye and SiNPs, high hydrophilicity and good water dispersion, excellent stability, and easy surface modification (5, 14). These high-quality characteristics indicate that colored SiNPs are good optical nano-labels to amplify the response signal in immunochromatographic strips (15–17).

*Brucella* spp. is a type of intracellular parasitic and facultative anaerobic Gram-negative short bacilli. According to the different susceptible hosts, *Brucella* spp. can be divided into six classical types, namely, *Brucella abortus, Brucella melitensis, Brucella ovis, Brucella canis, Brucella neotomae*, and *Brucella suis* (18). At the beginning of the twenty-first century, several novel species were identified, such as *Brucella pinnipedialis* (isolated from seals) and *Brucella ceti* (isolated from dolphins and whales) (19), as well as *Brucella microti*, which was isolated and identified from wild rats for the first time. Then, a new strain of *B. microti* was isolated from red foxes and soil in 2008 (20), *Brucella inopinata* was isolated from a breast-transplanted patient in 2010 (21), and another new strain of *Brucella* was isolated from frogs in 2012 (22). The discovery of these new *Brucella* spp. reveals that the natural host range of *Brucella* spp. has gradually expanded to non-human primates and amphibians. Among the abovementioned strains, *B. abortus, B. melitensis, B. neotomae*, and *B. suis* are human pathogenic bacteria, and *B. melitensis* has the strongest pathogenicity. According to the statistics of *B. melitensis* strains isolated from 29 provinces in China from 1950 to 1991, the proportion of *B. melitensis* strain type 1 and type 3 was 53.8 and 33.2%, respectively. However, since 1991, the proportion of type 3 strain has increased to 80% (23). Brucellosis, the disease caused by *Brucella* spp., can be transmitted from infected animals to humans, which brings particular threat to the people engaged in animal husbandry in some rural areas. The data from the World Health Organization demonstrate that brucellosis has affected more than 170 countries or regions in the world, and more than 400,000 new cases were reported every year (24). The main clinical manifestations of brucellosis include wave fever, night sweats, joint pain, abortion, infertility, and easy recurrence. The course of brucellosis varies from 1 month to more than a few years. Therefore, patients with brucellosis will face great economic burden and mental problems if they cannot be diagnosed or treated in time. On the contrary, most patients with brucellosis will recover within 3–6 months if brucellosis can be diagnosed in time. At present, the detection technology for brucellosis, such as bacterial culture and PCR, usually requires a relatively long time, high cost, expensive equipment, and complex operating procedures. However, brucellosis commonly occurs in farms and rural areas with poor facilities. The existing diagnosis technologies for brucellosis require professional testing personnel and equipment, which are not suitable for rural areas. Therefore, it is of great significance to establish a simple, rapid, and inexpensive diagnosis method for brucellosis for early diagnosis and timely treatment.

As little work using blue SiNPs as a reporter of LFIA to detect human brucellosis has been reported, here, an LFIA with a rapid, sensitive, and alternative diagnostic procedure for human brucellosis with a high degree of accuracy was developed based on blue SiNPs, *Staphylococcal* protein A (SPA), and surface lipopolysaccharide of *Brucella* spp. (LPS). At first, the precursor of blue SiNPs such as C.I. Reactive Blue 21 and colorless SiNPs was synthesized, and then C.I. Reactive Blue 21 was introduced onto the surface of colorless SiNPs in a given of amount of coupling substance to prepare blue SiNPs, followed by a layer of silica shell, which was formed by TEOS hydrolysis that was wrapped outside of SiNPs to avoid dye leakage. Afterwards, the amino group, which aims to combine with SPA by covalent bonding, was introduced to the surface of core-shell blue SiNPs *via* 3-aminopropyl triethoxysilane (APTES). After that, glutaraldehyde was used to activate the amino groups on the surface of blue SiNPs, and then SPA was added into above solution to obtain the blue SiNPs@SPA. The blue SiNPs@SPA as capture signal to catch the antibody in the brucellosis-positive serum. When SPA was combined with the antibodies in the brucellosis-positive serum, it was captured by LPS on the test line, forming an antigen–antibody sandwich structure, resulting in the T line turning blue. On this basis, the feasibility of this detection in human serum was also verified. We also discussed the feasibility of this work in human serum, and the results showed that it is acceptable to use blue SiNPs as visible labels of LFIA to diagnose human brucellosis in standard brucellosis serum and real human serum samples. Above all, a fast, one-step strategy and high-specificity method based on blue SiNPs for the diagnosis of human brucellosis has been developed, which can be applied in developing countries and rural areas.

## Materials and Methods

### Materials and Reagents

SPA and goat anti-mouse IgG were provided by Beijing Bioss Biotechnology Co., Ltd. and (3-Aminopropyl) triethoxysilane (APTES) was provided by Shanghai Aladdin Bio-Chem Technology Co., LTD. Glutaraldehyde was obtained from Sigma-Aldrich (Shanghai, China); bovine serum albumin (BSA) and tetraethyl orthosilicate (TEOS) were obtained from Shanghai Macklin Biochemical Co., Ltd. Anhydrous ethanol was obtained from Tianjin Xinbote Chemistry Co., Ltd. (Tianjin, China). Twenty five percent ammonia was purchased from Xilong Science Co., Ltd. Reagents used in indirect ELISA were purchased from Shanghai Jianglai Biotechnology Co., Ltd; phosphate buffered saline (PBS) buffers (pH 7.4) were obtained from Sangon Biotechnology Co., Ltd. (Shanghai, China). The glass fiber, releasing pad, nitrocellulose membrane, absorbing pad, and polyvinyl chloride sheet were obtained from Shanghai Jinbiao Biotechnology Co., Ltd. (Shanghai, China).

### Preparation of Core-Shell Blue SiNPs

Colorless SiNPs were synthesized by the Stöber method with a slight modification. A Stöber system containing 30 ml of anhydrous ethanol, 4 ml of water, and 5 ml of ammonia water was hydrolyzed for 30 min at 60°C. Then, 5 ml of TEOS was added slowly within 30 min, and the reaction continued for another 2 h. The final colorless SiNPs were isolated by centrifugation (7,000 rpm, 5 min) and finally freeze-dried by a Lyophilizer.

Dye-coated SiNPs were prepared by following a previous report with a slight modification (25). Briefly, 100 mg of colorless SiNPs was dissolved in 15 ml of water. After that, 20 μl of APTES and 100 μl (100 mg/ml) of C.I. Reactive Blue 21 aqueous solution were mixed with 15 ml of water, which contained 0.1 g SiNPs and was left to react for 2.5 h. Then, the precipitate was centrifugally collected and washed with ethanol and water.

A Stöber system that contained 10 ml of ethanol, 815 μl of H_2_O, 0.2 ml of TEOS, and 0.1 ml of NH_3_·H_2_O was hydrolyzed for 20 min, and then 5 ml of the above blue SiNPs was poured into the above solution and reacted for 12 h. After the reaction, the core-shell blue SiNPs were centrifuged and dispersed in pure water.

### Preparation of Amino-Modified Core-Shell Blue SiNPs

One hundred milligrams of core-shell blue SiNPs was dissolved in 20 ml of ethanol under ultrasonic conditions at room temperature for 30 min. After that, the above prepared core-shell blue SiNPs were transferred into a three-necked flask containing 80 ml of ethanol, and 100 μl of APTES was added and refluxed at 60°C for 7 h. The resulting amino-modified blue SiNPs were washed four times with water.

### Preparation of SPA-Coated Core-Shell Blue SiNPs

The SPA was combined with core-shell blue SiNPs through covalent crosslinking with a slight modification (26); 2.5 ml of glutaraldehyde and 9 ml of PBS (pH 7.4) were added into the above amino-modified core-shell blue SiNPs and then stirred for 2 h at room temperature to obtain glutaraldehyde-activated blue SiNPs. After that, 1 ml (1 mg/ml) of SPA was added into the above solution with 5 ml of PBS (pH 7.4) and then stirred for another 2 h. The resulting SPA-coated blue SiNPs were collected by centrifugation and dispersed in 10 ml of water with 1% BSA for 1 h at room temperature to block unreacted sites.

### Characterization of Synthesized SiNPs and Core-Shell Blue SiNPs

SiNPs and core-shell blue SiNPs were characterized by a transmission electron microscope (TEM) (200 kV) and a Fourier infrared transform spectrometer (FT-IR). KBr was set as the scanning background, and wavenumbers were set to 500–3,500 cm^−1^ of FT-IR. After the dry samples were mixed with KBr, the samples were scanned at a speed of 2 cm^−1^.

### Fabrication of Core-Shell Blue SiNPs-Based LFIA

**Scheme 1:**
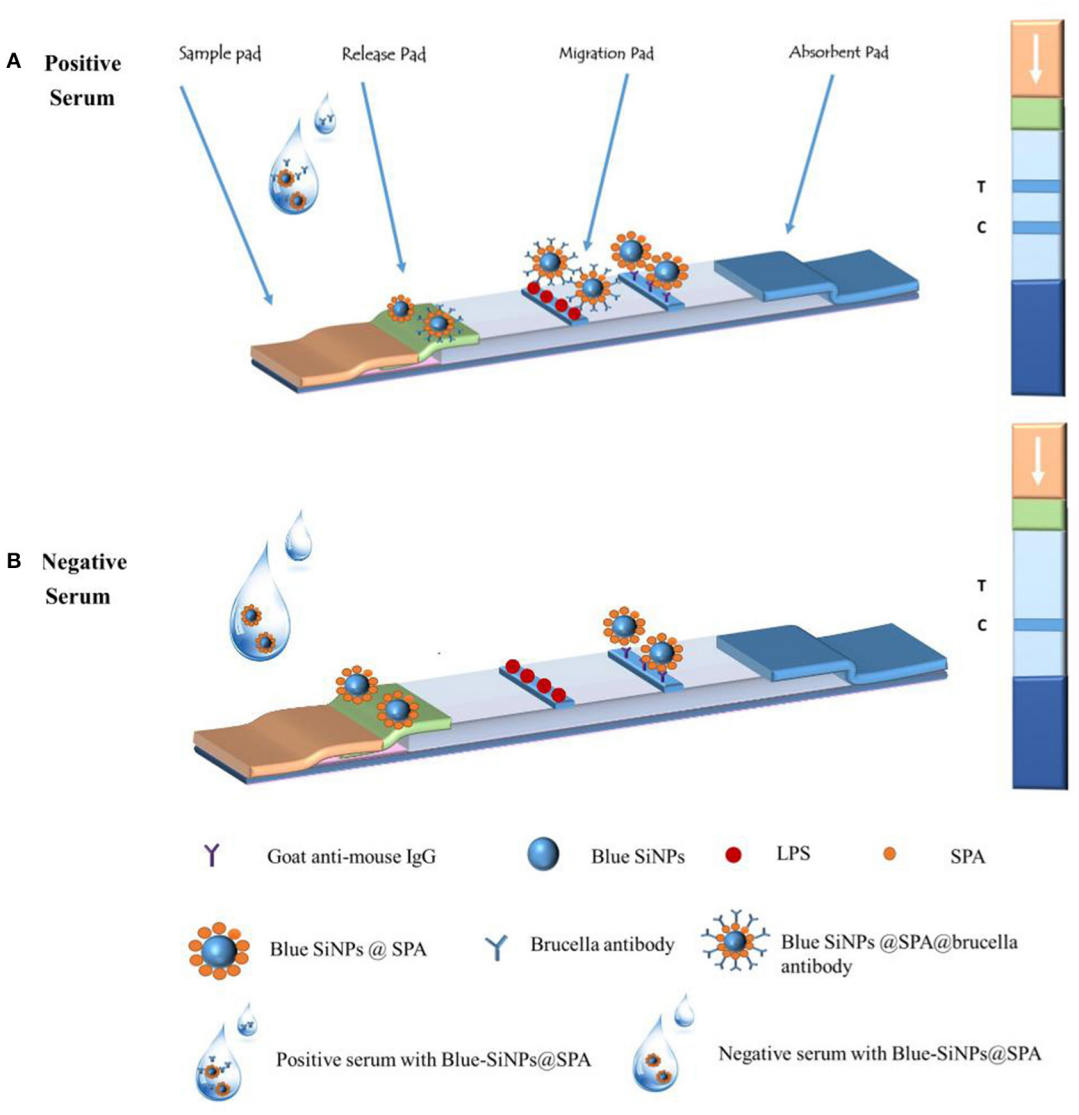
The principle of the core-shell blue SiNPs-based LFIA system and the illustration of the result judgment.

The structure of the core-shell blue SiNPs-LFIA is shown in Scheme 1. LPS (1 mg/ml) and goat anti-mouse IgG (0.6 mg/ml) were loaded on the T line and the control line (C line) separately. The distance between the two pipelines was 5 mm. Finally, NC membrane, releasing pad, adsorbent pad, and sample pad were fabricated onto the backing pad. The LFIA was stored in a dark and dry place.

### Brucellosis Serum Samples

Standard brucellosis serum (*B. melitensis*), brucellosis-positive control serum (*B. melitensis*), and negative control serum were purchased from Shanghai Jianglai Biotechnology Co., Ltd. The Brucellosis Prevention and Control Base, Chinese Centers for Disease Control and Prevention (Baicheng, Jilin, China) donated a total of 102 human serum samples from their three clinical groups, including 69 human brucellosis serum samples, 24 negative control serum samples from healthy individuals, and 9 serum samples infected with other bacteria. All the serum samples were stored in a −80°C refrigerator. The serum agglutination test (SAT), the gold standard diagnostic method for brucellosis, was run following the Health Industry Standards of the People's Republic of China: Diagnosis for brucellosis GB/T (WS 269-2019), and each sample was run with three parallel tests. Relevant information on the serum samples is presented in Supplementary Table 1.

### Application of LFIA in the Diagnosis of Brucellosis

Fifty microliters of human serum sample was fully mixed with 50 μl of blue SiNPs, and then the mixed solution was added dropwise to the sample pad of the strip. After 15 min of reaction, the visual results of the T line and C line were observed by the naked eye. As shown in Scheme 1, when the serum was a brucellosis-positive serum, the blue SiNPs@SPA bound with the *Brucella* spp. antibody in the serum, and then the blue SiNPs@SPA with antibody was captured by LPS on the T line, so the T line turned blue; some blue SiNPs@SPA that did not bind with the *Brucella* spp. antibody were captured by the secondary antibody on the C line, making the C line appear blue. When the serum was a brucellosis-negative serum, there was no *Brucella* spp. antibody in the serum; then, the blue SiNPs@SPA was not specifically captured by LPS on the T line, making it appear colorless, while the blue SiNPs@SPA was captured by the secondary antibody on the C line, making the C line appear blue.

To test the specificity of this method, nine non-brucellosis serum samples infected with other bacteria were used: *Escherichia coli O157:H7* serum, *Staphylococcus aureus* serum, *Staphylococcus epidermidis* serum, *Staphylococcus saprophyticus* serum, *Streptococcus salivarius* serum, *Streptococcus anginosus* serum, *Klebsiella pneumoniae* serum, *Salmonella enteritidis* serum, and *Ralstonia picri* serum.

To test the sensitivity of this method, standard brucellosis serum was diluted with saline in the range of 10^−1^ to 10^−6^ IU/ml to test the detection limit based on the methods we developed.

### The Comparison Between Our Developed LFIA and Indirect ELISA (IELISA)

In order to verify the effectiveness of the detection method that we developed, we compared the detection results of human serum samples between LFIA and iELISA. The SAT, the gold standard diagnostic method for brucellosis, was run following the Health Industry Standards of the People's Republic of China: Diagnosis for brucellosis GB/T (WS 269-2019), and each sample was run with three parallel tests. iELISA was run according to the previous protocol.

## Results and Discussion

### FT-IR Spectra of Blue SiNPs and Blue SiNPs-NH_2_

**Figure 1:**
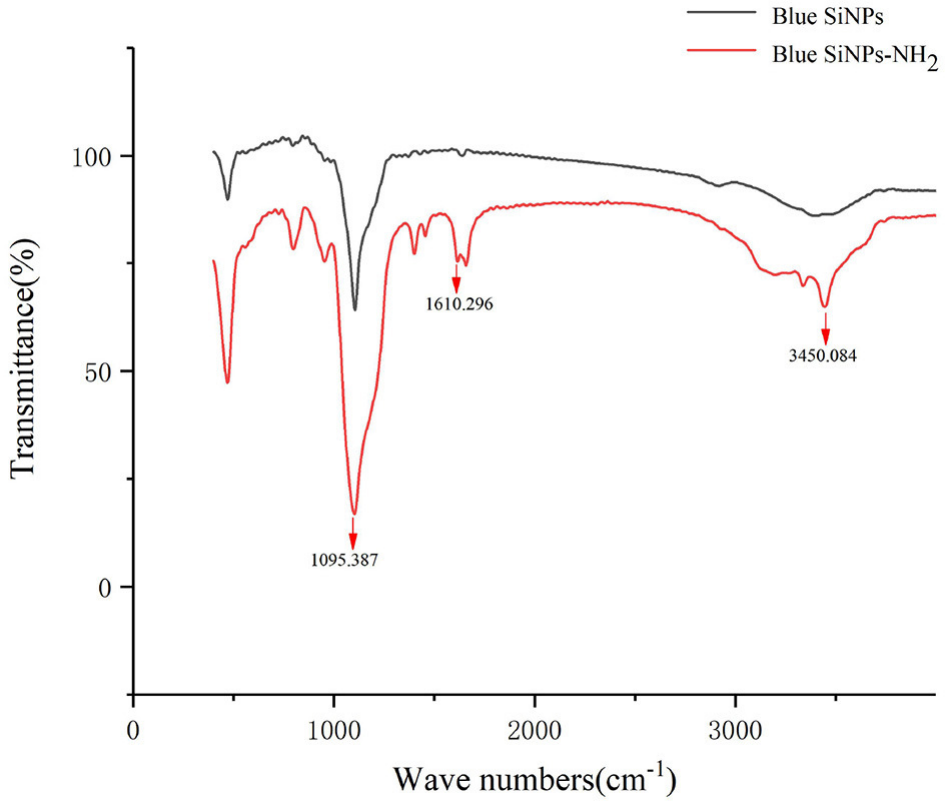
FT-IR spectra of blue SiNPs (top) and blue SiNP-NH_2_ (bottom).

FT-IR was used to characterize whether the amino group was modified on the surface of SiNPs. FT-IR provided direct proof for the functionalization of amino groups on the SiNPs, because FT-IR can detect the stretching vibration of chemical bonds on the surface of blue SiNPs and blue SiNPs-NH_2_, as shown in Figure 1. Both blue SiNPs and blue SiNPs-NH_2_ showed strong infrared absorption bands in the region of 800–1,180 cm^−1^, corresponding to Si-O-Si on the silica surface. As shown in the FT-IR absorption spectra of blue SiNPs-NH_2_, 3,450 and 1,610 cm^−1^ were attributed to the N-H bonds of the amino group. The above results were comparable with the blue SiNPs being successfully wrapped with an amino-modified silica shell; therefore, it is reasonable to believe that APTES was successfully conjugated with blue SiNPs, and amino groups were successfully coupled on blue SiNPs.

### Characterization of Core-Shell Blue SiNPs

**Figure 2:**
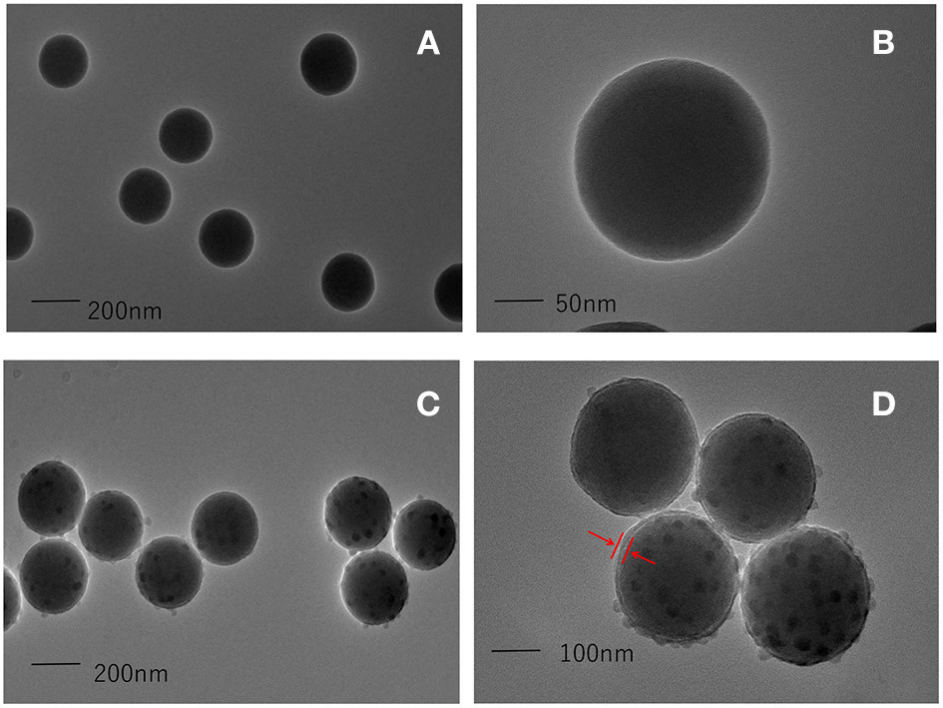
TEM images of colorless SiNPs **(A,B)**, and core-shell blue SiNPs **(C,D)**. The red arrow means that a layer of silica shell has been successfully wrapped on the outside of the silica nanoparticles, and the position of the shell is marked with the red arrow.

A TEM method was used to characterize the synthesized SiNPs. The TEM images of the colorless SiNPs and core-shell blue SiNPs are shown in Figures 2A,B. Under the same magnitude of enlargement, both the SiNPs and core-shell blue SiNPs showed good morphology, dispersion, and dispensability. According to the TEM images (Figures 2C,D), there was a layer of silica shell that can be seen on the surface of SiNPs (The part indicated by the red arrow). Moreover, in the process of coating SiNPs, many small uneven particles were formed on the surface of the silica shell by self-hydrolysis of TEOS. According to previous literature (17), the unsmooth surface of the particles can provide more binding sites for protein and help to coat more SPA. Therefore, the results of TEM showed that blue SiNPs were successfully prepared in this work.

Also, we tested the steadiness of blue SiNPs compared with colloidal gold nanoparticles in different pH values and different concentrations of salt solution (Supplementary Figure 1). The results showed that the scalar variability of pH and concentrations of NaCl solution had little influence on the change in blue SiNPs' color; on the contrary, aggregated colloidal gold nanoparticles demonstrated a different color with the varying pH and concentration of NaCl solution, indicating that blue SiNPs were more stable than colloidal gold nanoparticles at different pH values and concentrations of the NaCl solution. These results indicated that blue SiNPs were more suitable as visible labels of LFIA than colloidal gold nanoparticles due to their good stability.

### Characterization of SPA-Conjugated Core-Shell Blue SiNPs

LFIA was employed to identify that SPA-conjugated core-shell blue SiNPs were successfully synthesized. The results could be observed by the naked eye, and it was obvious that the SPA-conjugated core-shell blue SiNPs were captured by a secondary antibody, and it showed a strong blue color at the C line (Supplementary Figure 2B). In contrast, the core-shell blue SiNPs could not combine with a secondary antibody, so the C line was colorless (Supplementary Figure 2A). This result demonstrated that SPA was successfully combined with core-shell blue SiNPs.

### Optimization of Experiment Conditions

#### Optimization of the Dosage of Dye

To achieve the optimal response condition, four factors that would affect the final results were investigated. First of all, the amount of dye used in the synthesis of blue SiNPs was optimized. When too much dye was used, it was dissolved in the solvent and made it difficult for the blue SiNPs to be coated on the shell, which had been hydrolyzed by TEOS; however, if only a small amount of dye was used, then the color was not bright enough. So, it is necessary to find a suitable amount of dye that can not only make the color of blue SiNPs bright enough, but also prevent interferences. From Supplementary Figure 3, the results indicated that when the additional amount of dye was 100 μl (100 mg/ml), the color of blue SiNPs was bright enough and dye did not leak too much. Therefore, we decided to set the final additional amount of dye at 100 μl.

#### Optimization of the Dosage of LPS

Secondly, the sprayed concentration of LPS on the T line was optimized. It was obvious that the concentration of LPS affected the binding ability of LPS with antibodies in standard brucellosis serum. It can be seen from Supplementary Figure 4 that when the concentration of LPS was 1 mg/ml, the binding ability of LPS with standard brucellosis serum was better than the other concentrations of LPS. Therefore, 1 mg/ml was chosen as the optimum concentration of LPS.

#### Optimization of the Dosage of Goat Anti-mouse IgG

Next, we optimized the dosage of goat anti-mouse IgG. The sprayed concentration of goat anti-mouse IgG on the C line of the test strip could affect the binding ability of goat anti-mouse IgG with SPA. The goat anti-mouse IgG was printed in different concentrations (1, 0.8, 0.6, 0.4, and 0.2 mg/ml) on the control line. It can be seen from Supplementary Figure 5 that when the concentration of goat anti-mouse IgG was 0.6 mg/ml, the color of the C line was bright enough, and the binding ability between goat anti-mouse IgG and SPA was better. In order to save experimental materials, 0.6 mg/ml was chosen as the best concentration of goat anti-mouse IgG.

#### Optimization of the Dosage of SPA

Finally, we optimized the dosage of SPA. The concentration of SPA that coated on the surface of blue SiNPs could also affect the binding ability between SPA and antibodies in standard brucellosis serum. Experimental studies on the binding ability between SPA and LPS under different concentrations of SPA were carried out, and it can be seen from Supplementary Figure 6 that when the concentration of SPA was 1 mg/ml, the binding ability was better than the other concentrations. Therefore, 1 mg/ml was chosen as the suitable concentration of SPA.

### Sensitivity of Core-Shell Blue SiNPs-Based LFIA

**Figure 3:**
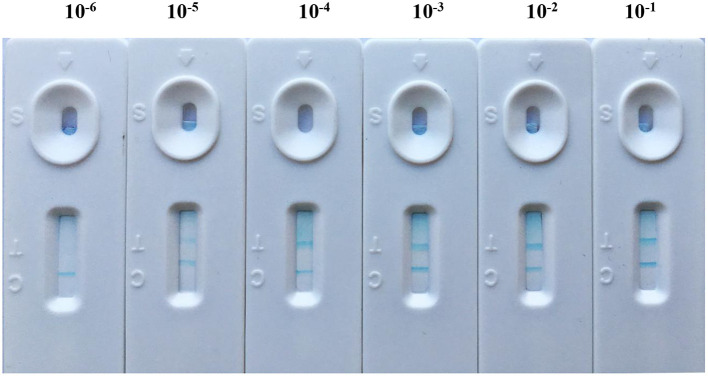
The sensitivity of LFIA based on blue SiNPs for visible detection of standard brucellosis serum, after a series of standard brucellosis-positive serum (standard brucellosis-positive serum was diluted to 10^−1^ to 10^−6^ IU/ml) were mixed with blue SiNPs.

After the abovementioned experiment condition optimization procedure, the core-shell blue SiNPs-based LFIA was evaluated by standard brucellosis serum under optimal conditions. After the series of standard brucellosis serum (standard brucellosis serum was diluted to 10^−1^ to 10^−6^ IU/ml) were mixed with blue SiNPs, the sensitivity of the core-shell blue SiNPs-based LFIA for *Brucella* antibody was obtained. From Figure 3, we can see that when the serum dilution was 10^−5^, the T line still showed a blue color. According to the above results, we can conclude that our LFIA could detect *Brucella* spp. antibody at a level of 10^−5^ (0.01 IU/ml) in serum with no false positives.

### Specificity of Core-Shell Blue SiNPs-Based LFIA

**Figure 4:**
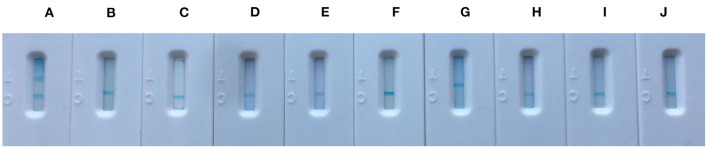
The specificity picture from left to right was **(A)** positive brucellosis serum, **(B)** *Staphylococcus epidermidis* serum, **(C)** *Streptococcus anginosus* serum, **(D)** *Escherichia coli O157:H7* serum, **(E)** *Klebsiella pneumoniae* serum, **(F)** *Staphylococcus aureus* serum, **(G)** *Staphylococcus saprophyticus* serum, **(H)** *Ralstonia picri* serum, **(I)** *Salmonella enteritidis* serum, and **(J)** *Streptococcus salivarius* serum.

The specificity of core-shell blue SiNPs-based LFIA was evaluated with control experiments that involved *S. epidermidis* serum, *S. anginosus* serum, *E. coli O157:H7* serum, *K. pneumoniae* serum, *S. aureus* serum, *S. saprophyticus* serum, *R. picri* serum, *S. enteritidis* serum, and *S. salivarius* serum. To explore the specificity of LFIA based on blue SiNPs to diagnose human brucellosis-positive serum, the nine abovementioned serums, as a control group, were studied. After incubation, we saw that the T line and C line of the strip both displayed a bright blue color in the presence of human brucellosis-positive serum; in contrast, we did not observe a blue color on the T line in the presence of the other tested serums (Figure 4). These results indicated the excellent specificity of our testing strip based on the core-shell blue SiNPs for the detection of human brucellosis serum.

### Detection of Human Serum Samples

In order to evaluate the sensitivity and accuracy of our method, we applied our developed method to detect human serum samples. A total of 102 human serum samples were firstly detected by the serum agglutination test (SAT) to determine whether the serum could be diagnosed as positive or negative; SAT followed the Health Industry Standards of the People's Republic of China: Diagnosis for brucellosis GB/T (WS 269-2019) to guarantee that there were no false positives, and each sample was run with three parallel tests to ensure accuracy. Indeed, the LFIA that we developed could detect antibodies in serum, in the whole blood, and even in plasma. Usually in a typical LFIA, the complex matrix can make the NC membrane change colors, which leads to a high background, especially in whole blood. However, due to the bright color of the blue SiNPs, even if the NC membrane turns red, the results were also easy to observe. In particular, the SiNPs can combine with a variety of dyes and appear in a variety of colors; therefore, the SiNPs can be more widely applied in the detection technology.

**Figure 5:**
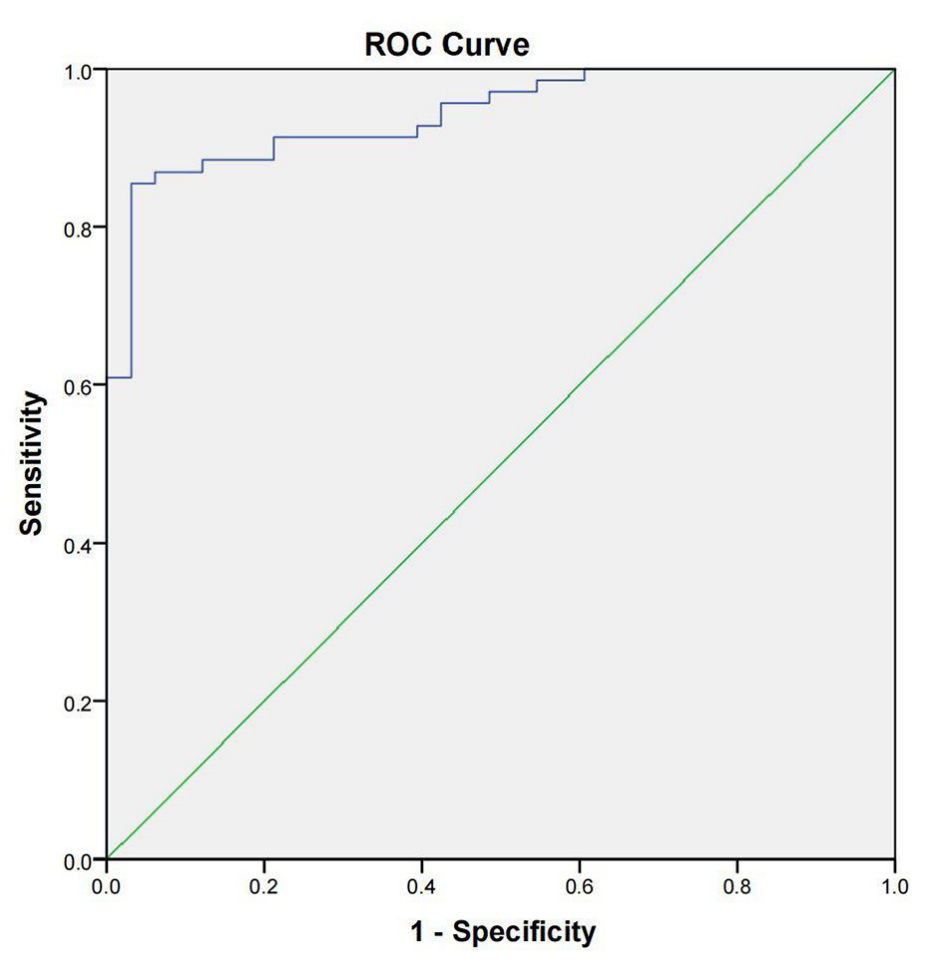
The receiver operating characteristic curve of the lateral flow immunoassay with the SAT method as state variable. The area under the curve (AUC) was 0.942 (95% CI, 0.900–0.984); the standard errors (under the non-parametric assumption) were 0.022, *p* < 0.05.

We applied our developed LFIA to detect 69 positive brucellosis serum samples, 24 negative brucellosis serum samples, and nine non-brucellosis serum samples infected with other bacteria samples, and all the serum samples were verified by SAT. The sensitivity, specificity, and accuracy of core-shell blue SiNPs-based LFIA were assessed completely. From the results, we can see that with our method, 61 positive brucellosis serum samples were identified as “positive” correctly, eight positive brucellosis serum samples were wrongly identified as “negative,” 2 negative serum samples were wrongly identified as positive, and 31 of the negative samples were identified as “negative” correctly, as described in Table 1. Also, we used SPSS software to draw a receiver operating characteristic (ROC) curve (Figure 5) to evaluate the feasibility of our strip for actual samples; area under the curve (AUC) was used to present the accuracy of the method. The AUC of our experiment was 0.942 [95% confidence interval (CI), 0.900 to 0.984], demonstrating that the accuracy of this method is convincing and our detection results are highly authentic. In addition, a positive predictive value (PPV) of 87.0% and a negative predictive value (NPV) of 93.9% of our developed method were obtained from an optimum cutoff value of 68.94 (Youden's index optimum). With this cutoff value, 61 of 69 brucellosis samples were screened correctly as positive, and only two negative cases were screened incorrectly as positive.

**Table 1:** The evaluation results of our developed LFIA and iELISA.

**Methods**	**Cutoff value**	**Positive**		**Negative**		**PPV (%)**	**NPV (%)**
		**TP**	**FN**	**TN**	**FP**		
LFIA	≥68.94	61	2	31	8	87.0	93.9
iELISA	≥0.532	59	1	32	10	85.5	96.9

*SAT was used as the gold standard to distinguish the results of LFIA and iELISA. TP, true positive; TN, true negative; FP, false positive; FN, false negative; PPV, positive predictive value, TP/(TP+FP) × 100%; NPV, negative predictive value, TN/(TN+FN) × 100%*.

**Figure 6:**
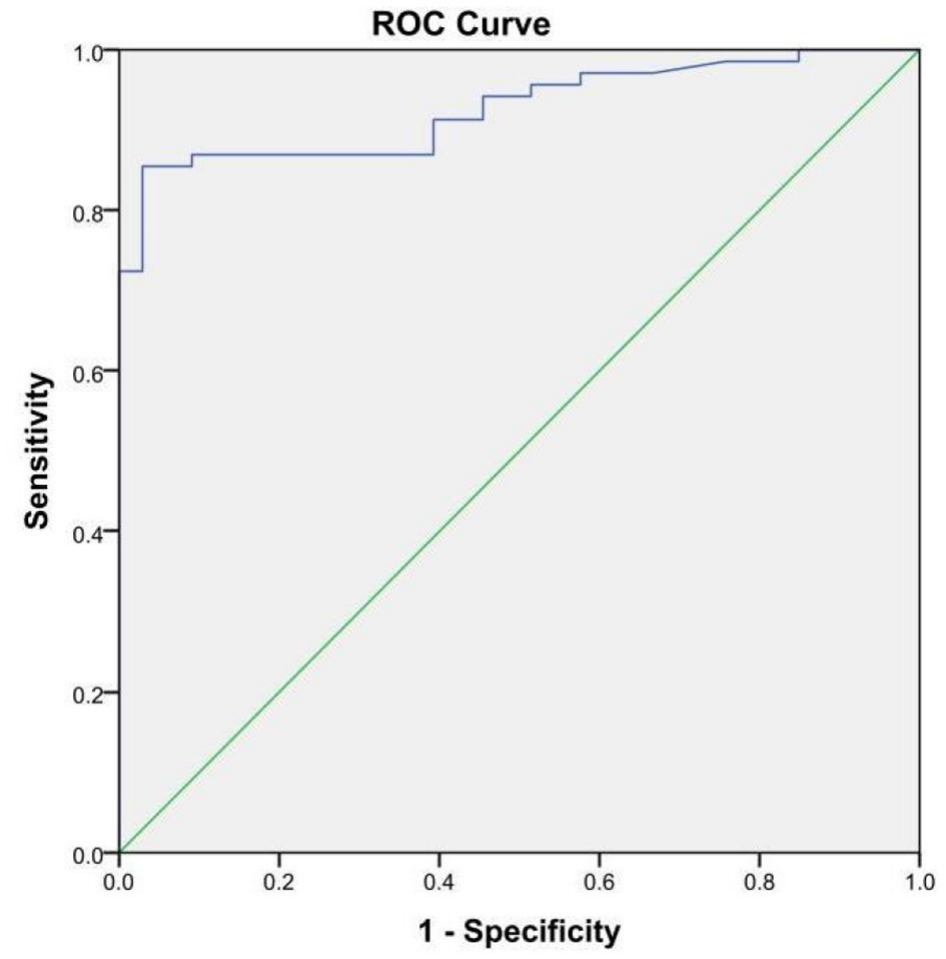
The receiver operating characteristic curve of the indirect ELISA with the SAT method as state variable. The area under the curve (AUC) was 0.926 (95%CI, 0.877–0.975); the standard errors (under the non-parametric assumption) were 0.025, *p* < 0.05.

Furthermore, we also compared our methods with iELISA to evaluate the sensitivity and specificity. Compared with our developed method, iELISA was also used to detect the same 102 serum samples, and the resulting ROC curve and the results of iELISA are shown in Figure 6 and Table 1. From Figure 6, we can see that the AUC of the ROC curve was 0.926 (95% CI, 0.877–0.975). When the optimal cutoff value was selected at 0.532 (Youden's index optimum) as the standard critical value of diagnosis, the PPV was 85.5% and the NPV was 96.9%. With this cutoff value, 59 of 69 brucellosis samples were screened correctly as positive, and only one negative case was screened incorrectly as positive. Above all, there was no significant difference in the diagnostic sensitivity and specificity between our developed LFIA and iELISA (Table 1). Furthermore, the sensitivity of our experiment was slightly better than iELISA. These results indicated that the accuracy and sensitivity of our method were reliable, and our developed method can be applied in the diagnosis of human brucellosis in the future.

## Discussion and Conclusion

As a new nanomaterial, SiNPs have significant advantages, namely, stability and feasibility, compared with other nanoparticles. Based on their stability, bioavailability, colorfulness, sensitivity, and specificity, SiNPs have shown great promise in immunology (27), virus detection (28), cell biology (29), and environmental monitoring (30). Blue SiNPs as a new nanomaterial were used as a signal probe and SPA was used as a capture probe to catch and immobilize antibodies in the serum as well as combined with the secondary antibody on the C line in our research. When SPA-functionalized blue SiNPs (the capture probe) were obtained, the capture probe could catch the antibody when the serum was brucellosis-positive, and then the captured antibody specifically bound with LPS on the T line, which made the T line blue. The probe we synthesized had better stability and sensitivity compared with other traditional capture probes. As the signal probe, the SiNPs were coated with dye and then were wrapped with a silica shell hydrolyzed by TEOS, demonstrating its environmentally friendly and simplified procedure. The usage of SPA made the detection more accurate and simple. SPA can be linked to the Fc fragment of IgG molecules by hydrophobic interactions, resulting in the orientation and arrangement of antibody molecule probes on the surface of blue SiNPs. This oriented fixation is better organized than either direct physical adsorption or covalent binding, and it has less impact on the activity of antibodies. Thus, the entire activity of the functionalized capture probe has been improved (4, 31). Above all, a LFIA-based visible blue SiNPs label was developed for the rapid screening or diagnosis of people who have suffered from human brucellosis. As a promising probe, blue SiNPs can be successfully applied in the detection of human brucellosis by LFIA. Compared with the traditional diagnosis methods, the LFIA based on blue SiNPs can not only save time but also reduce cost simultaneously; in addition, blue SiNPs have many advantages such as good stability, bright color, and lower price, compared to other traditional methods. Taken together, these results suggest that the LFIA based on the blue SiNPs can be applied to diagnose brucellosis in remote areas where there is a lack of testing condition and instruments. Moreover, the minimize visual cutoff values of the antibody titer in serum was 0.01 IU/ml, indicating the high sensitivity of our method. Additionally, there was no cross-reaction between different serum samples when we applied the strip to human serum samples from different individuals infected with various microorganisms. In summary, the blue SiNPs-based LFIA has shown great promise for rapid and inexpensive diagnosis of human brucellosis. Moreover, this LFIA-based detection method may have future application for multiple immunochromatography detection, which deserves further development. As far as our current experiment is concerned, a single color cannot be satisfied with multiple detection. In the future, we will also develop various colored SiNPs for multiple immunochromatography detection.

## Data Availability Statement

The original contributions presented in the study are included in the article/Supplementary Material, further inquiries can be directed to the corresponding authors.

## Ethics Statement

The studies involving human participants were reviewed and approved by Institutional Research Ethics Committee of Medicine, the School of Public Health, Jilin University, permit number: JLU2014-0303.2.4. The patients/participants provided their written informed consent to participate in this study.

## Author Contributions

LG, JL, and KX conceived and designed the study. LG, DW, FL, and YZ performed the assay of experimental detection. YW, JZ, and LZ analyzed the data. LG drafted the manuscript. JL, JW, and XS provided constructive opinions and suggestions. KX, JL, and XS reviewed and made improvements in the manuscript. All authors have read and approved the final version of this manuscript.

## Funding

This work was financially supported by the Department of Education of Jilin Province, China (Grant Numbers: JJKH20211221KJ, JJKH20180239KJ and JJKH20211220KJ), the National Natural Science Foundation of China (Grant Numbers: 82173572 and 81401721), the Science and Technology Development Bureau of Jilin Province, China (Grant Number: 2018010195JC), the Jilin Provincial Development and Reform Commission (Grant Number: 2020C038-7), the Jilin Provincial Health and Family Planning Commission (Grant Number: 2017J074), and the Graduate Innovation Fund of Jilin University (Grant Number: 101832020CX275).

## Conflict of Interest

The authors declare that the research was conducted in the absence of any commercial or financial relationships that could be construed as a potential conflict of interest.

## Publisher's Note

All claims expressed in this article are solely those of the authors and do not necessarily represent those of their affiliated organizations, or those of the publisher, the editors and the reviewers. Any product that may be evaluated in this article, or claim that may be made by its manufacturer, is not guaranteed or endorsed by the publisher.
