# Supplementary material for: Lateral Flow Immunoassay for Visible Detection of Human Brucellosis Based on Blue Silica Nanoparticles
Source: Front Vet Sci. 2021 Dec 3;8:771341. doi: 10.3389/fvets.2021.771341 (PMC8677672; doi:10.3389/fvets.2021.771341)
Supplement: Supplementary file 1 [file Data_Sheet_1.doc]

Lateral Flow Immunoassay Based on Blue Silica Nanoparticles for Visible Detection of Human Brucellosis

Lirui Ge^1,2^, Dan Wang^1,2^, Fengnan Lian^1,2^, Jinbin Zhao^1,2^, Yue Wang^1,2^, Yuyi Zhao^1,2^, Lanting Zhang^1^, Juan Wang^1^, Xiuling Song^1^, Jinhua Li^1^*, Kun Xu^1,2^*

^1^*School of Public Health, Jilin University, Changchun, 130021, China*

*^2^* *Public Health Detection Engineering Research Center of Jilin Province, Changchun, 130021, China*

*Corresponding author at: School of Public Health of Jilin University, Changchun, Jilin, 130021, P. R. China.

1. mail: [xukun@jlu.edu.cn](mailto:xukun@jlu.edu.cn) & [jinhua1@jlu.edu.cn](mailto:jinhua1@jlu.edu.cn)

**pH: 2 3 4 5 6 7 8 9 10 11 12**

**A**


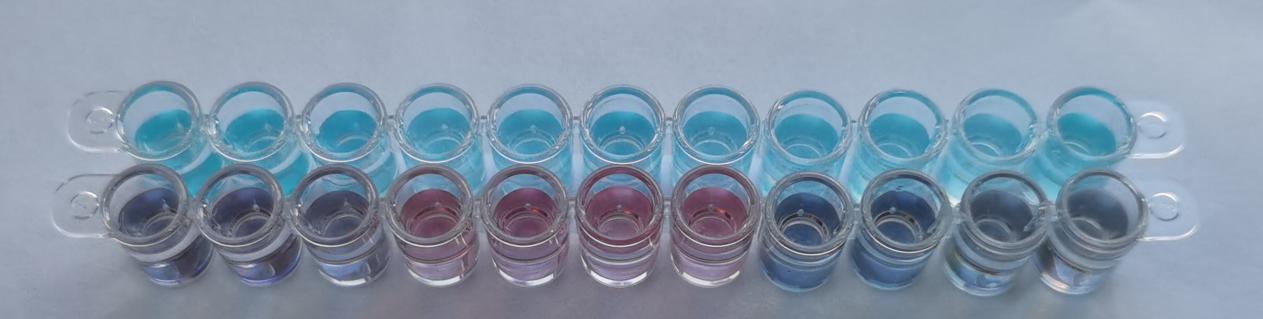


**NaCl: 0.25% 0.5% 1% 2% 3% 4% 5%**

**B**

**
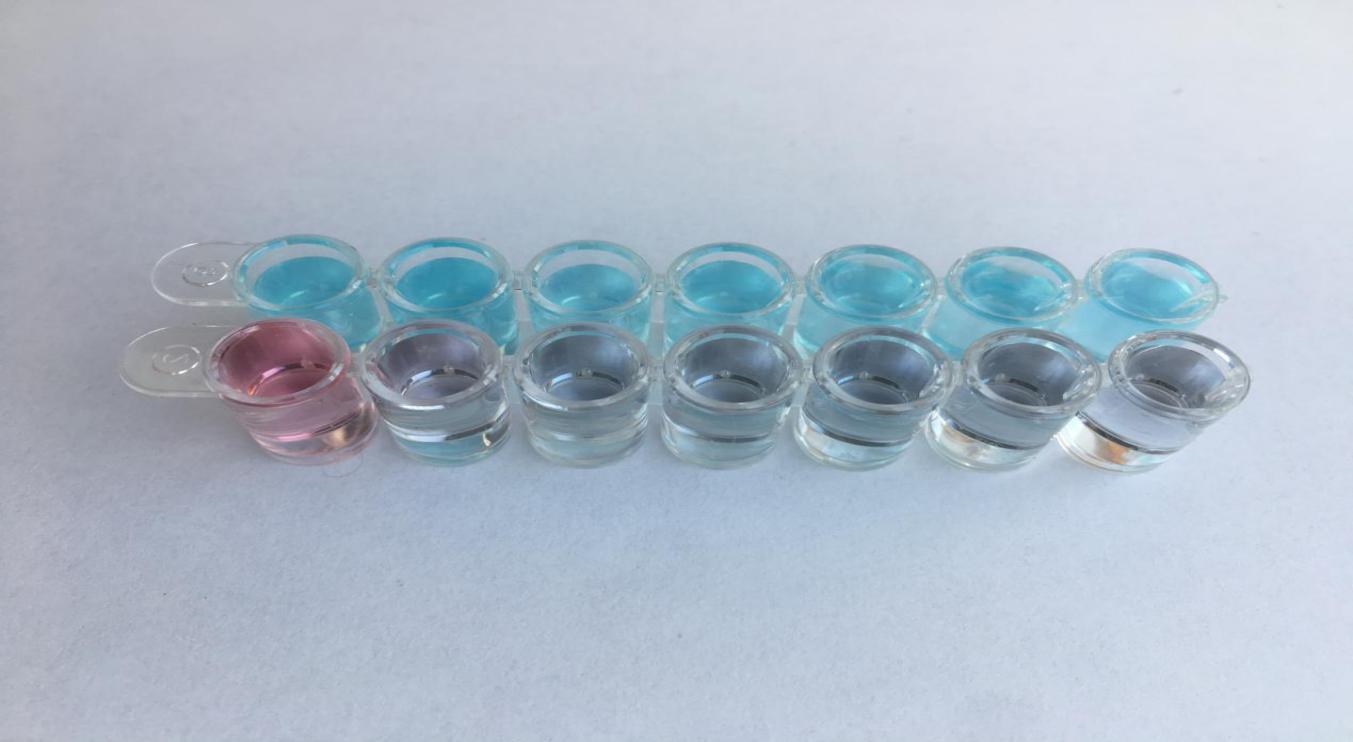
**

**Fig S1.** The steadiness photograph of core-shell blue SiNPs solution (up) and

colloidal gold solution (bottom), when they were dispersed in different pH

(A) and different concentrations of NaCl (B) solutions.


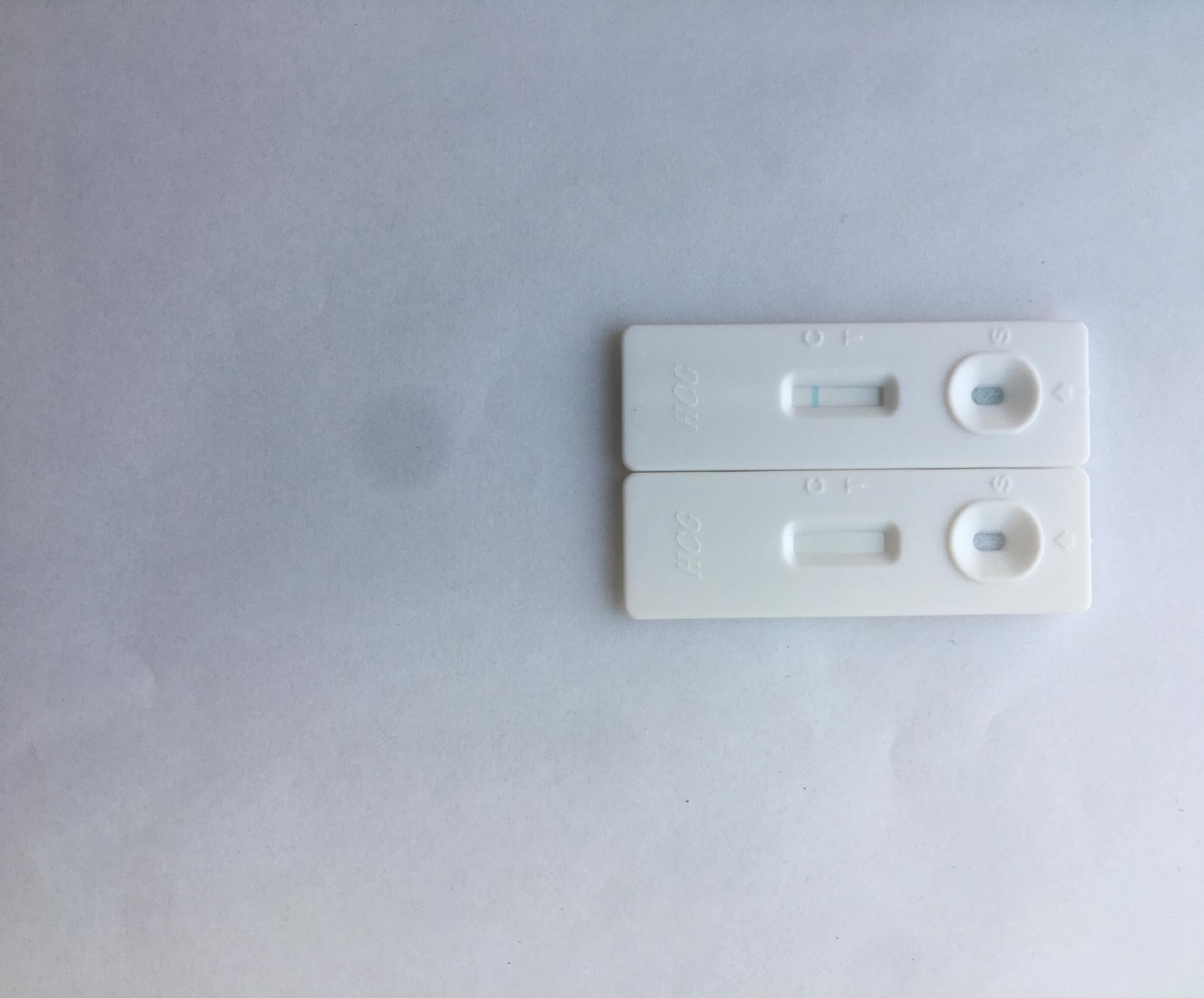


**A**

**B**

**Fig S2.** The comparison of LFIA made by bare core-shell blue SiNPs (A) and

SPA-coated core-shell blue SiNPs (B).

**300 µL 250 µL 200 µL 150 µL 100 µL**


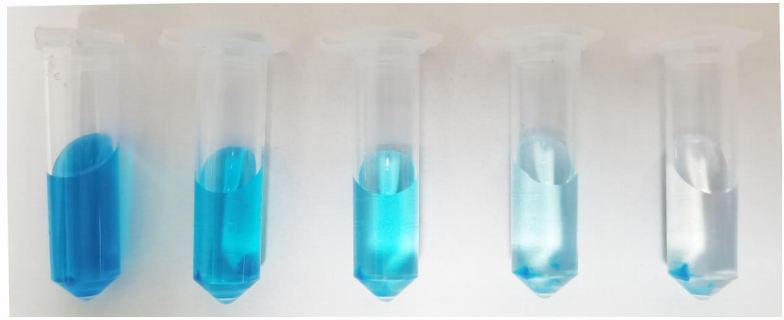


**Fig S3.** The images of corresponding dye leakage between different addition of dye with SiNPs.

**1.2 mg/mL 1.0 mg/mL 0.8 mg/mL 0.6 mg/mL 0.4 mg/mL**


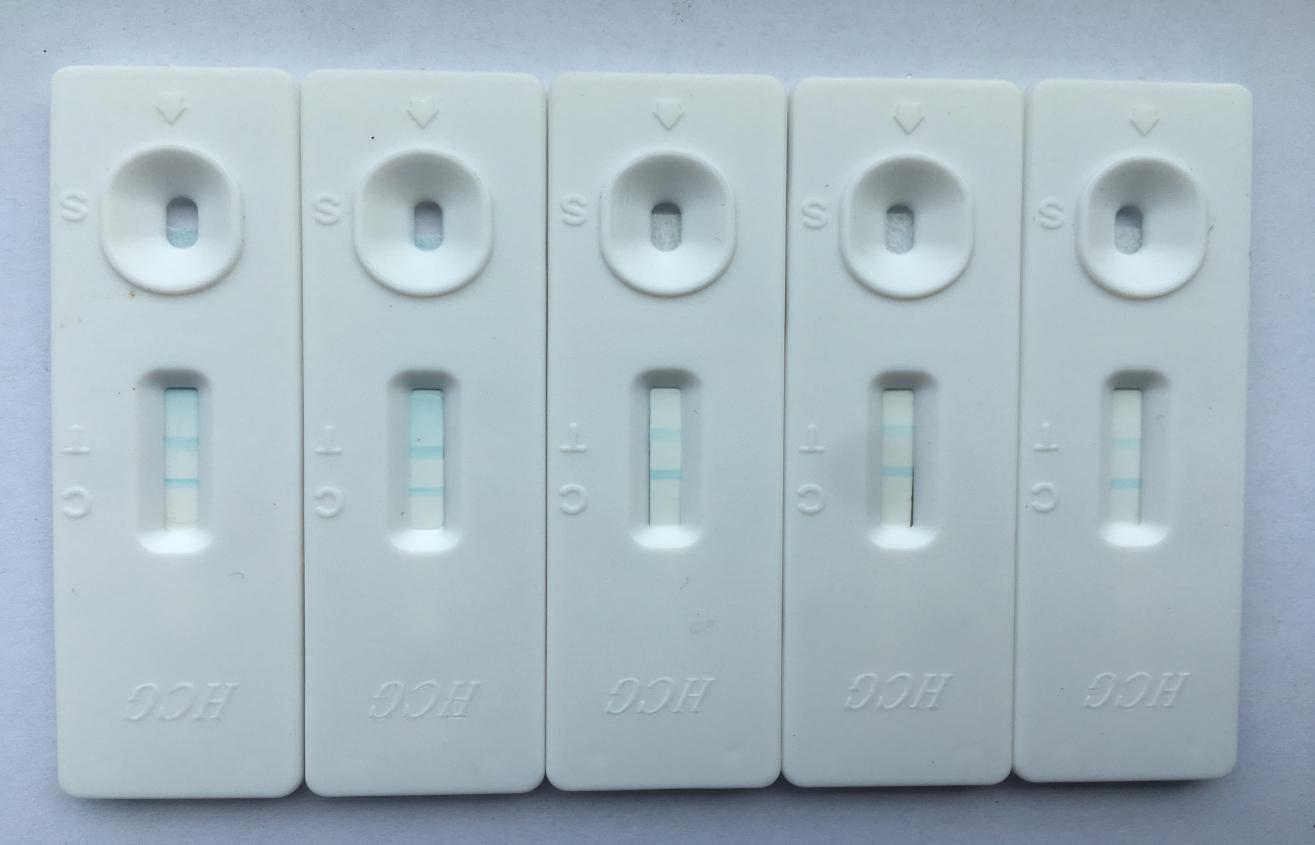


**Fig S4.** The corresponding binding ability between different addition of LPS with *Brucella* antibody.

**1.0 mg/mL 0.8 mg/mL 0.6 mg/mL 0.4 mg/mL 0.2 mg/mL**


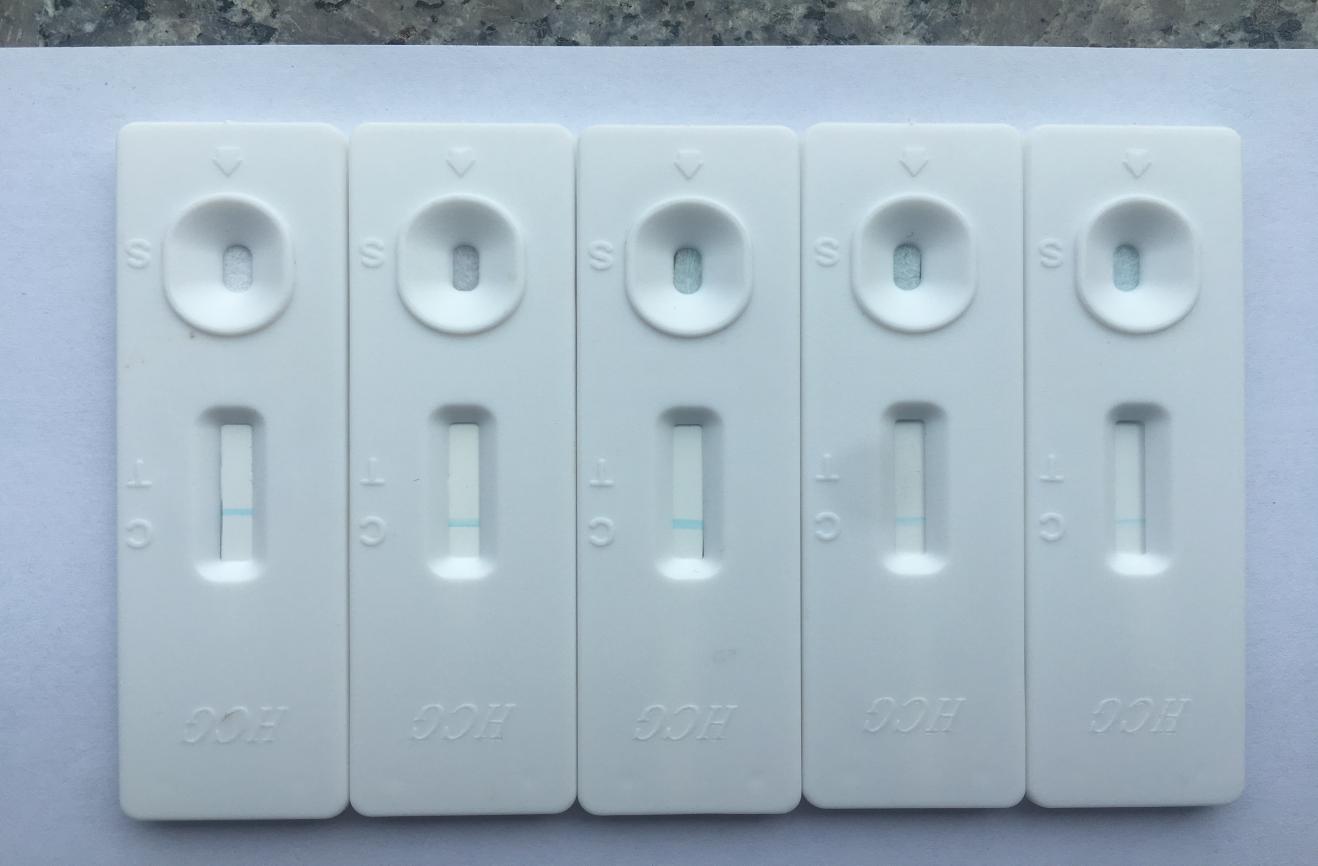


**Fig S5.** The corresponding binding ability between different addition of goat anti-mouse IgG with SPA.

**0.2 mg/mL 0.4 mg/mL 0.6 mg/mL 0.8 mg/mL 1.0mg/mL**


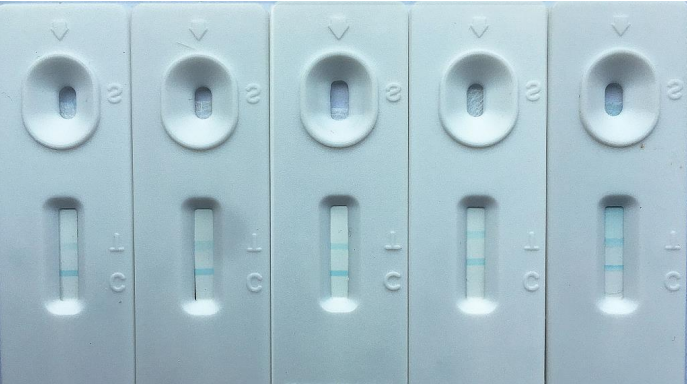


**Fig S6.** The addition of SPA and their corresponding binding ability with LPS.

**Table S1. The titer of *Brucella* antibodies in 102 human sera samples by SAT**

| **sample No.** | titer | **sample No.** | titer |
| --- | --- | --- | --- |
| **1** | 1：320 | **34** | 1：160 |
| **2** | 1：640 | **35** | 1：1280 |
| **3** | 1：640 | **36** | 1：1280 |
| **4** | 1：640 | **37** | 1：2560 |
| **5** | 1：640 | **38** | 1：20480 |
| **6** | 1：640 | **39** | 1：1280 |
| **7** | 1：40 | **40** | 1：1280 |
| **8** | 1：640 | **41** | 1：1280 |
| **9** | 1：640 | **42** | 1：1280 |
| **10** | 1：640 | **43** | 1：1280 |
| **11** | 1：640 | **44** | 1：1280 |
| **12** | 1：640 | **45** | 1：640 |
| **13** | 1：2560 | **46** | 1：2560 |
| **14** | 1：5120 | **47** | 1：2560 |
| **15** | 1：5120 | **48** | 1：20480 |
| **16** | 1：10240 | **49** | 1：2560 |
| **17** | 1：160 | **50** | 1：2560 |
| **18** | 1：10240 | **51** | 1：2560 |
| **19** | 1：10240 | **52** | 1：1280 |
| **20** | 1：2560 | **53** | 1：1280 |
| **21** | 1：5120 | **54** | 1：1280 |
| **22** | 1：2560 | **55** | 1：1280 |
| **23** | 1：1280 | **56** | 1：2560 |
| **24** | 1：10240 | **57** | 1：640 |
| **25** | 1：10240 | **58** | 1：2560 |
| **26** | 1：320 | **59** | 1：640 |
| **27** | 1：10240 | **60** | 1：20480 |
| **28** | 1：320 | **61** | 1：20480 |
| **29** | 1：10240 | **62** | 1：5120 |
| **30** | 1：5120 | **63** | 1：2560 |
| **31** | 1：2560 | **64** | 1：2560 |
| **32** | 1：160 | **65** | 1：2560 |
| **33** | 1：2560 | **66** | 1：2560 |
| **67** | 1：2560 | **89** | <1:20 |
| **68** | 1：640 | **90** | <1:20 |
| **69** | 1：640 | **91** | 1:40 |
| **70** | <1:20 | **92** | <1:20 |
| **71** | <1:20 | **93** | <1:20 |
| **72** | <1:20 | **94** | <1:20 |
| **73** | <1:20 | **95** | <1:20 |
| **74** | <1:20 | **96** | <1:20 |
| **75** | <1:20 | **97** | <1:20 |
| **76** | 1:40 | **98** | <1:20 |
| **77** | <1:20 | **99** | 1:40 |
| **78** | <1:20 | **100** | <1:20 |
| **79** | <1:20 | **101** | <1:20 |
| **80** | <1:20 | **102** | <1:20 |
| **81** | <1:20 |  |  |
| **82** | <1:20 |  |  |
| **83** | <1:20 |  |  |
| **84** | <1:20 |  |  |
| **85** | <1:20 |  |  |
| **86** | <1:20 |  |  |
| **87** | <1:20 |  |  |
| **88** | <1:20 |  |  |
